# Neuroligin-2 Expression in the Prefrontal Cortex is Involved in Attention Deficits Induced by Peripubertal Stress

**DOI:** 10.1038/npp.2015.200

**Published:** 2015-08-05

**Authors:** Stamatina Tzanoulinou, Clara García-Mompó, Orbicia Riccio, Jocelyn Grosse, Olivia Zanoletti, Panagiotis Dedousis, Juan Nacher, Carmen Sandi

**Affiliations:** 1 Department of Life Sciences, Laboratory of Behavioral Genetics, Brain Mind Institute, Ecole Polytechnique Fédérale de Lausanne, Lausanne, Switzerland; 2 Neurobiology Unit and Program in Basic and Applied Neurosciences, Cell Biology Department, Universitat de València, Valencia, Spain; 3 Department of Informatics, University of Piraeus, Piraeus, Greece; 4 CIBERSAM: Spanish National Network for Research in Mental Health, Madrid, Spain; 5 Fundacion Investigacion Hospital Clinico de Valencia, INCLIVA, Valencia, Spain

## Abstract

Emerging evidence indicates that attention deficits, which are frequently observed as core symptoms of neuropsychiatric disorders, may be elicited by early life stress. However, the mechanisms mediating these stress effects remain unknown. The prefrontal cortex (PFC) has been implicated in the regulation of attention, including dysfunctions in GABAergic transmission, and it is highly sensitive to stress. Here, we investigated the involvement of neuroligin-2 (NLGN-2), a synaptic cell adhesion molecule involved in the stabilization and maturation of GABAergic synapses, in the PFC in the link between stress and attention deficits. First, we established that exposure of rats to stress during the peripubertal period impairs attention in the five-choice serial reaction time task and results in reductions in the GABA-synthesizing enzyme glutamic acid decarboxylase in different PFC subregions (ie, prelimbic (PL), infralimbic, and medial and ventral orbitofrontal (OFC) cortex) and in NLGN-2 in the PL cortex. In peripubertally stressed animals, NLGN-2 expression in the PL and OFC cortex correlated with attention measurements. Subsequently, we found that adeno-associated virus-induced rescue of NLGN-2 in the PFC reverses the stress-induced attention deficits regarding omitted trials. Therefore, our findings highlight peripuberty as a period that is highly vulnerable to stress, leading to the development of attention deficits and a dysfunction in the PFC GABAergic system and NLGN-2 expression. Furthermore, NLGN-2 is underscored as a promising target to treat stress-induced cognitive alterations, and in particular attentional deficits as manifested by augmented omissions in a continuous performance task.

## Introduction

Attention deficits lie at the core of cognitive dysfunctions manifested in different neuropsychiatric disorders (Barnett *et al*, 2010; Millan *et al*, 2012). Early life stress is a known risk factor for the development of psychopathologies (Heim and Nemeroff, 2001), and emerging clinical (Chugani *et al*, 2001; Stevens *et al*, 2008) and preclinical (Carlyle *et al*, 2012; Comeau *et al*, 2014; Fuentes *et al*, 2014) evidence indicates that it can trigger attentional problems. However, the potential mechanisms mediating attentional deficits induced by exposure to early stress remain largely unknown.

The prefrontal cortex (PFC) is one of the key brain regions implicated in the regulation of attentional processes (Robbins, 2002; Rossi *et al*, 2009) and is highly susceptible to stress (Arnsten, 2009; Liston *et al*, 2006). Converging evidence highlights alterations in the function of GABAergic transmission in the PFC as a relevant mechanism underlying attentional deficits: (i) the inhibition of GABA_A_ receptors in the PFC leads to attentional deficits (Enomoto *et al*, 2011; Paine *et al*, 2011; Pehrson *et al*, 2013; Pezze *et al*, 2014) and (ii) a reduced GABAergic tone, including a reduction in the GABA-synthesizing enzyme glutamic acid decarboxylase (GAD) (Akbarian *et al*, 1995; Volk *et al*, 2000), was found in the PFC in schizophrenia, a disorder with core attentional deficits.

Early life stress has been reported to induce long-lasting GABAergic changes in several brain regions (Jacobson-Pick *et al*, 2008; Martisova *et al*, 2012; Tzanoulinou *et al*, 2014a, 2014b). Importantly, stress applied at adulthood was found to alter the expression levels of several cell adhesion molecules (Sandi, 2004; Sandi and Bisaz, 2007), including neuroligin-2 (NLGN-2) (van der Kooij *et al*, 2014), which is located at inhibitory GABAergic synapses and plays a key role in their regulation (Chih *et al*, 2005; Huang and Scheiffele, 2008; Sudhof, 2008). NLGN-2 belongs to the family of NLGN postsynaptic cell adhesion molecules, which regulate different aspects of synaptic function (Levinson and El-Husseini, 2005; Sudhof, 2008). However, the possibility that NLGN-2 expression is persistently altered by early life stress has not been addressed.

Here, we investigated whether alterations in the expression of NLGN-2 and GAD in the PFC are associated with attentional deficits programmed by early life stress. We used a rat model of peripubertal (comprising childhood and puberty) stress-induced psychopathology (Marquez *et al*, 2013; Toledo-Rodriguez and Sandi, 2011), as the functional maturation of local prefrontal GABAergic circuits occurs during this developmental period (Caballero *et al*, 2014; Thomases *et al*, 2013). Attention was evaluated in the five-choice serial reaction time task (5-CSRTT), which is the rodent equivalent of the continuous performance task in humans (Dalley *et al*, 2008; Robbins, 2002). Furthermore, we determined the expression levels of NLGN-2 and GAD in different PFC regions and investigated the causal involvement of NLGN-2 in stress-induced attention deficits using a rescue experiment utilizing viral-induced NLGN-2 overexpression (OE).

## Materials and Methods

### Animals

Subjects were the offspring of Wistar Han rats (Charles River Laboratories, France), bred in our animal facility. They were kept on a 12 h light–dark cycle (lights on at 0700 hours). Food and water were available *ad libitum*, except for the food-deprivation period during the 5-CSRTT, when animals were kept to 85–90% of their free-feeding weight. All procedures were conducted in conformity with the Swiss National Institutional Guidelines on Animal Experimentation and approved by a license from the Swiss Cantonal Veterinary Office Committee for Animal Experimentation.

### Experimental Design

At weaning (P21), male rats from different litters were distributed into different home cages in groups of two to three non-siblings, and each cage was randomly assigned to control (CTRL) or peripubertal stress (PPS) conditions. At P28, the stress protocol began (Marquez *et al*, 2013). At adulthood (P90), animals were handled for 3 consecutive days and subsequently either allocated to behavioral experiments (14 CTRL and 16 PPS animals that were subjected to the 5-CSRTT) or maintained under basal conditions and perfused for immunohistochemical analyses (9 CTRL and 6 PPS). Two days after the last behavioral testing, all rats were killed, and their brains were rapidly extracted, frozen in cold isopentane and stored at −80 °C until further processing. For the NLGN-2 OE study, an additional cohort of adult CTRL and PPS animals was subjected to surgery and virus infusion at adulthood, yielding the following four groups: CTRL-empty (*N*=9), CTRL-NLGN-2 OE (*N*=12), PPS-empty (*N*=10), and PPS-NLGN-2 OE (*N*=10).

### Peripubertal Stress Protocol

PPS was performed as previously described (Marquez *et al*, 2013; Veenit *et al*, 2014) and involved exposure to two fear-inducing stressors (the synthetic fox odor trimethylthiazoline (Phero Tech, Delta, BC, Canada) administered in a plastic box (38 × 27.5 × 31 cm) and exposure to an elevated platform (12 × 12 cm)) presented in a variable schedule in the span of 7 days across the P28–P42 period. Each stressor lasted for 25 min. For more information, see Supplementary Materials and Methods.

### Five-Choice Serial Reaction Time Task (5-CSRTT)

The 5-CSRTT requires the rats to allocate their limited attentional resources on randomly presented stimuli (spatially divided attention). The experiment was simultaneously performed in six animals using six testing boxes (Coulbourn Instruments, Bilaney Consultants, Germany). Each box consists of two aluminum walls, one of which is curved and equipped with five circular holes, whereas the opposite wall is equipped with a food well connected to a pellet dispenser. All of the circular holes and the food magazine are equipped with infrared beams crossing their entrance to detect the animals' responses. A previously described protocol (Bari *et al*, 2008) was used with minor modifications. Briefly, following habituation to the apparatus, rats were required to learn to nose poke into five randomly illuminated holes within a limited time period to obtain one sucrose pellet (45 mg, Bio-Serv, Flemington, NJ, USA). The rats received 5 daily 30-min sessions per week, with a 100 pellet cut-off (whichever condition was met first). The training began with easy task contingencies in the early stages to facilitate learning and became progressively difficult throughout a total of 12 training stages. When the predetermined criteria for each stage were met, the animals were advanced to the next stage. When the animals were well-trained, ie, their behavior was stable for 6–10 sessions at the most advanced stage, the major 5-CSRTT parameters from the last 4 sessions were averaged, thus representing their baseline performance.

A response (ie, a nose poke) into one of the holes during the inter-trial interval (ITI) was considered a ‘premature response', whereas failure to respond was scored as an ‘omission'. ‘Accuracy' was calculated as the number of correct responses divided by the sum of correct and incorrect responses and is expressed as a percentage. ‘Continuous hits into the magazine' and ‘latencies' to respond correctly and to collect the reward were also estimated. The latency to collect the reward and the total number of trials were examined and served as indices of motivation to perform the task, as previously proposed (Amitai and Markou, 2010). ‘Total errors' were calculated as the sum of omission and commission errors (ie, incorrect pokes) and were divided by the total number of performed trials. In addition to these standard parameters, we calculated (per animal and per session in a trial-by-trial manner) the ‘standard deviation (SD) of the reaction time' (RT^SD^) for the 4 days that constituted the well-trained performance phase or the baseline. Furthermore, to study compulsivity behaviors in more detail, different variables were calculated. Specifically, an index of continuous hits into the magazine was defined as the immediate repetition of head entries, whereas a second index was defined as the number of additional pokes into the magazine following food delivery. Finally, the total number of trials and premature responses were subtracted from the total magazine hits within a session to obtain an additional generic index of unnecessary repeated responses in the food dispenser.

In addition, to assess the long-term effects of PPS on the acquisition of the 5-CSRTT, the initial period of training was also examined. Specifically, basic parameters of the 5-CSRTT, such as accuracy, omissions, and total errors, as well as the cumulative number of days to reach the next stage were investigated during the first 15 sessions. For statistical analyses, acquisition data were also analyzed in blocks of five consecutive sessions. At the most advanced stage of testing, the light stimulus in the holes lasted for 0.5 s with the ITI being 5 s. For the NLGN-2 OE experiment (see below), the light stimulus lasted 1 s and the ITI was 5 s. For additional details on the 5-CSRTT procedure, see the Supplementary Materials and Methods.

### Laser Capture Microdissection

Laser Capture Microdissection (LCM) was applied, as previously described (Timmer *et al*, 2011), on coronal sections of 20 μm aided by a Zeiss PALM laser system. The sections were put on membrane covered slides (Palm Microlaser Technologies, Carl Zeiss, Germany) and stored at −20 °C covered with RNAlater ICE (Ambion, TX, USA). Prior to LCM, the sections were stained with the HistoGene LCM Frozen Section Staining kit (Arcturus Bioscience, Switzerland). The orbitofrontal (OFC), prelimbic (PL), and infralibic (IL) cortex from both hemispheres were dissected, and the target tissue was placed in RNAase-free tubes and subsequently incubated in 100 μl of lysis buffer (RNAqueous Micro Kit, Ambion) for 30 min at 42 °C. After spinning down, the tissue was stored at −80 °C until further processing.

### Gene Expression Analysis

Total RNA from the regions dissected with LCM was isolated using the RNAqueous Micro kit and cDNA was synthesized using the Superscript VILO kit (Life Technologies). For quantitative RT–PCR, PCR reactions were performed in triplicate using SYBR Green PCR Master Mix (Applied Biosystems) in an ABI Prism 7900 Sequence Detection system (Applied Biosystems). Two genes were used as internal controls: gamma-actin (actg1) and eukaryotic elongation factor-1 (eef1). The primers for NLGN-2 (RefSeq: NM_053992.1) were designed using the Assay Design Center software from the Roche Applied Science (FW sequence: 5′-CCAAAGTGGGCTGTGACC-3′, RV sequence: 5′-CCAAAGGCAATGTGGTAGC-3′). Gene expression was analyzed with qBase 1.3.5 software using the comparative cycle threshold method.

### GAD-6 Immunohistochemistry and Quantification of Neuropil Immunoreactivity

To analyze whether PPS induced changes on the neuropil expression of GAD 65/67 (GAD-6), immunohistochemistry was performed as previously published (Tzanoulinou *et al*, 2014a). We analyzed PFC regions, namely the IL, PL (coordinates: bregma: 3.72 mm, interaural: 12.72 mm), cingulate 1 (Cg1), and Cg2 (bregma: 1.20 mm, interaural: 10.20 mm) regions of the medial PFC (mPFC), as well as the medial orbital (MO), ventral orbital (VO), lateral orbital (LO), and the dorsolateral orbital (DLO) part of the OFC cortex (bregma: 5.16 mm, interaural: 14.16 mm). For further information, see Supplementary Materials and Methods, and Supplementary Figure S4.

### Viral OE of NLGN-2

Naive CTRL and PPS rats were subjected to surgeries in the PFC after P90. After a 4-week period required for viral-induced NLGN2 OE, the animals were handled for 3 days before training in the 5-CSRTT started. The conditions for viral NLGN-2 OE were selected following pilot experiments and our previous work. Briefly, an adeno-associated AAV1/2 vector (http://www.genedetect.com) containing a CAG-HA-tagged-NLGN-2-WPRE-BGH-polyA-expression cassette was used (Figure 4a; GeneDetect, New Zealand). Half of the CTRL and PPS animals were injected with an empty construct (AAV1/2-CAG-Null/Empty WPRE-BGH-polyA). The coordinates to target the mPFC were +3.1 mm anterior, ±0.5 mm lateral, and −4.2 mm ventral, and to target the OFC +4.9 mm anterior, ±1 mm lateral, and −3.7 mm ventral to bregma, according to the atlas by Paxinos and Watson (1998). For further information, see Supplementary Materials and Methods.

### Statistics

Data were analyzed with Student *t*-tests, ANOVAs, and repeated measures ANOVAs, unless stated otherwise, using the statistical package SPSS 17.0 (Chicago, IL). Data is represented as the mean±SEM and significance set at *p*<0.05. Graphs were created using GraphPad Prism 5. For further details, see Supplementary Materials and Methods.

## Results

### Peripubertally Stressed Animals Exhibit Attention Deficits in the 5-CSRTT

**Figure 1:**
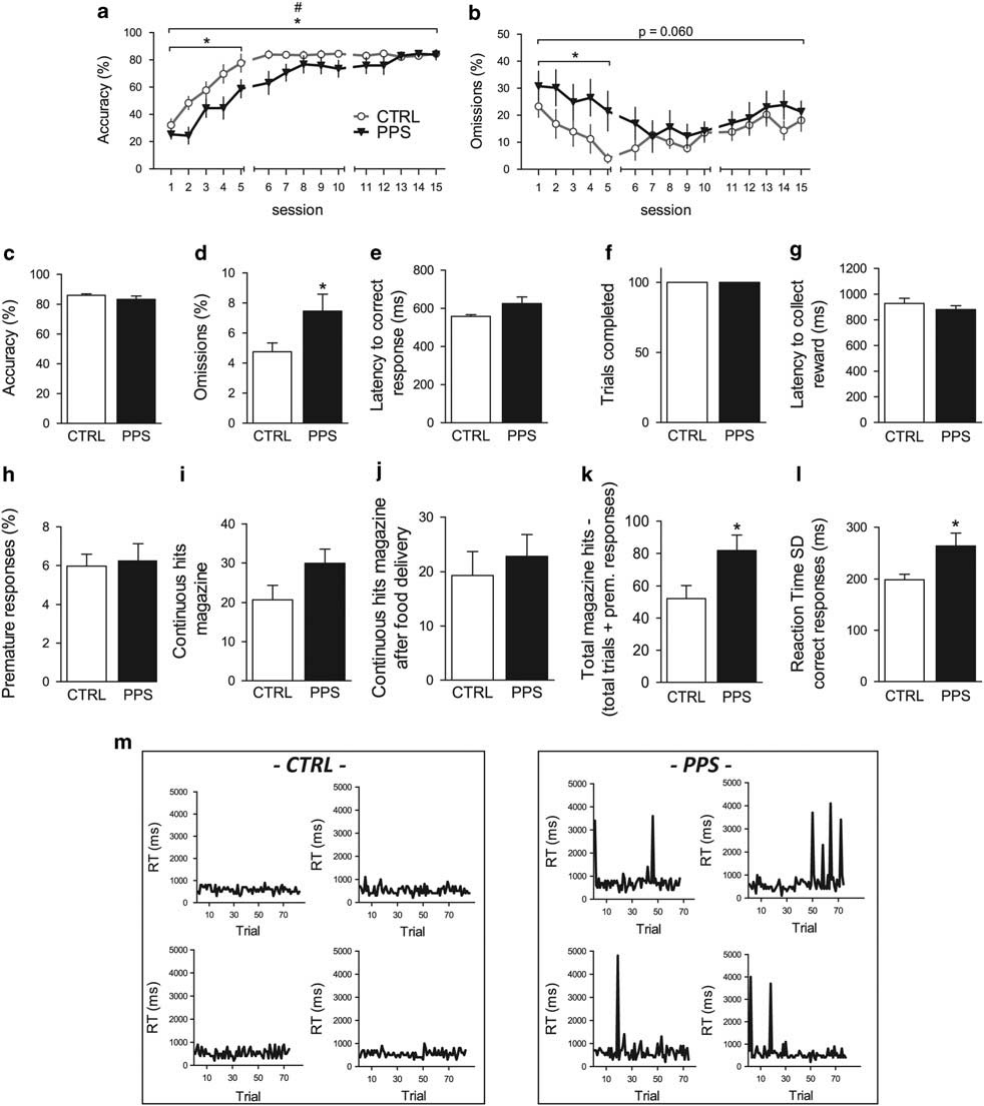
Effects of peripubertal stress exposure on 5-CSRTT performance at adulthood during acquisition (a and b) and during well-trained (baseline) conditions (c–m). During acquisition, peripubertally stressed (PPS) animals showed decreased accuracy, particularly during the first five training sessions (a), when they also showed more omissions (b). When tested under well-trained conditions, PPS animals did not exhibit altered accuracy (c) but they continued showing higher omissions than CTRLs (d). No significant differences were observed between PPS and CTRL rats in their latency to respond correctly (e) or in the number of trials completed (f) and in their latency to collect the reward (g). Furthermore, PPS animals did not show signs of impulsivity (h), while they showed a trend toward increased repetitive responding in the food magazine (i) but not following food delivery (j). However, when food dispenser head entries that ended either with the completion of a trial or with a premature response were subtracted from the total dispenser entries, PPS animals showed augmented magazine hits as compared with CTRLs (k). PPS animals showed increased intra-individual variability of reaction time for correct responses as measured by the average SD (l). Representative panels of reaction times for correct responses for CTRL (left) and PPS rats (right) (m). *N*: CTRL=14, PPS=16. *p<0.05, main effect of stress; ^#^*p*<0.05, stress × time interaction, results are expressed as the mean±SEM. 5-CSRTT, five-choice serial reaction time task; CSRTT, choice serial reaction time task; CTRL, control.

During training, PPS rats exhibited decreased accuracy in the 5-CSRTT compared with CTRL rats (Figure 1a) (main effect of stress: *F*_(1,28)_=4.470, *p*=0.044; stress × time interaction: *F*_(5,134)_=2.331, *p*=0.048), which was particularly manifested during the first block of five sessions (*F*_(1, 28)_=7.079, *p*=0.013). PPS animals also exhibited a trend toward increased omissions (Figure 1b) (main effect of stress: *F*_(1,28)_=3.827, *p*=0.060), which was significant for the first block of five sessions (*F*_(1,28)_=4.699, *p*=0.039). Moreover, PPS rats performed more total errors (*F*_(1,28)_=4.359, *p*=0.046) and required significantly more days to advance through the training stages (*χ*^2^_(1)_=4.326, *p*=0.038) than CTRL rats (see Supplementary Results and Supplementary Figure S1).

When performance was assessed under well-trained, ie, baseline conditions, PPS rats did not exhibit accuracy deficits (Figure 1c) (*t*_28_=1.087, *p*=0.286). However, PPS rats exhibited a higher number of omissions than CTRL rats (Figure 1d) (*t*_22_=−2.145, *p*=0.043). PPS rats also exhibited trends toward increased latency to respond correctly (Figure 1e) (*t*_28_=−1.789, *p*=0.084). No differences were observed between CTRL and PPS rats in the amount of trials completed (Figure 1f) (*t*_28_=0.933, *p*=0.359) or in their latency to collect the reward (Figure 1g) (*t*_28_=0.968, *p*=0.342), indicating the rats' motivation to perform the task. No significant difference was observed between PPS and CTRL rats in the number of premature responses (Figure 1h) (*t*_28_=−0.243, *p*=0.810). A trend toward increased continuous responses in the food magazine was observed for PPS animals (Figure 1i) (*t*_28_=−1.776, *p*=0.087) but not for continuous responses in the food magazine following food delivery (Figure 1j) (*t*_28_=−0.577, *p*=0.569). However, PPS animals exhibited increased head entries into the food magazine when the pokes that ended in the completion of a trial or in a premature response were subtracted from the total magazine nose pokes (Figure 1k) (*t*_28_=−2.302, *p*=0.029), possibly indicating an increase in compulsive responses.

In addition, we assessed the animals' intra-individual reaction time variability (IIV), which is a parameter that is frequently perturbed in neuropsychiatric disorders that involve attention deficits (Di Martino *et al*, 2008; Geurts *et al*, 2008; Kaiser *et al*, 2008; Klein *et al*, 2006). Remarkably, PPS animals exhibited significantly higher variability in their reaction times for correct responses (Figure 1l) (*t*_20_=−2.384, *p*=0.027). Figure 1m shows representative panels of reaction times for correct responses for CTRL and PPS rats, illustrating that the observed variability in PPS animals is due to an augmented number of trials with high reaction times (8.31% trials with RT above 1 s), whereas significantly lower percentages were observed in CTRLs (2.41% trials with RT above 1 s; *p*=0.012, Mann–Whitney test). No significant differences were observed in reaction time for incorrect trials (data not shown).

A principal component analysis of the aforementioned 5-CSRTT parameters indicated that PPS animals performed poorly compared with CTRL rats in a composite index of general performance (see Supplementary Materials and Methods, Supplementary Results, and Supplementary Figure S2). Finally, PPS animals exhibited similar number of premature responses as CTRLs on prolongation of the ITI, which is a methodological manipulation used to probe the animal's impulsive behavior (McNamara *et al*, 2010) (see Supplementary Materials and Methods, Supplementary Results, and Supplementary Figure S3).

### PPS Leads to Reduced GAD Immunoreactivity in the PFC

**Figure 2:**
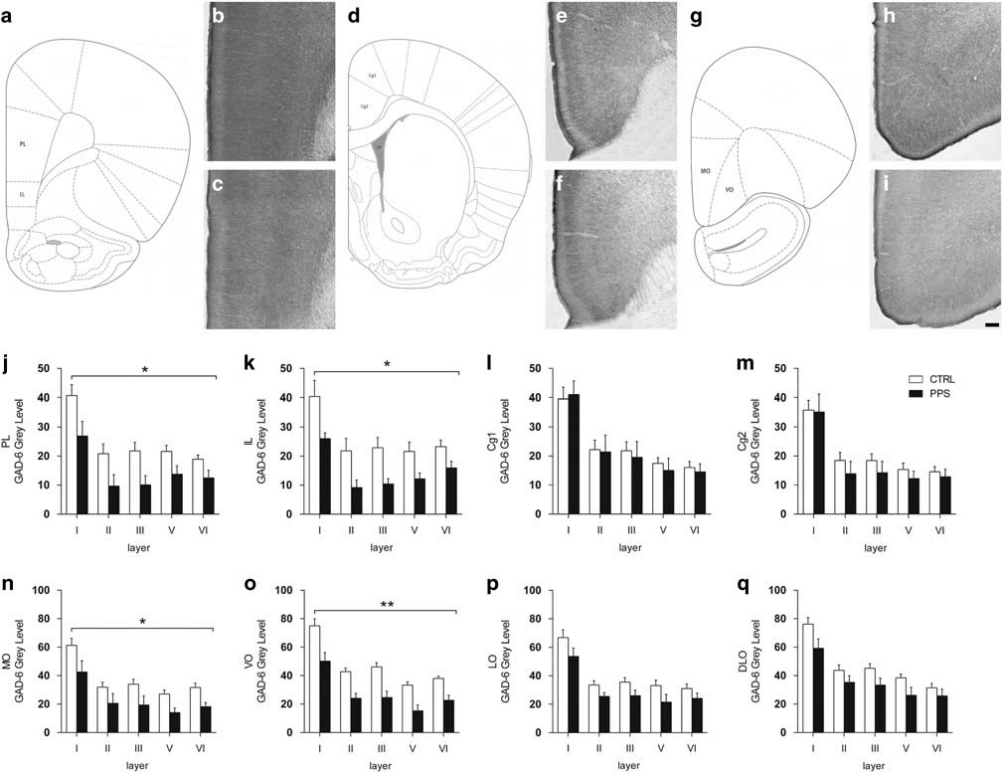
Effects of peripubertal stress on the expression of glutamic acid decarboxylase 65/67 (GAD-6) in the prefrontal cortex during adulthood. Schematic diagram of the prelimbic and infralimbic regions (a) and representative photomicrographs of these regions for CTRL (b) and PPS (c) animals. d depicts the cingulate cortex 1 and 2, while e and f depict representative images for the cingulate cortex from CTRL and PPS animals, respectively. The schematic diagram of the medial orbitofrontal and ventral orbitofrontal cortices is shown in g, and photomicrographs of the GAD-6 expression for these regions are depicted for CTRL (h) and PPS (i) animals. A significant decrease of GAD protein was observed across the layers of the prelimbic (j) and infralimbic (k) subregions of the medial PFC of PPS animals as compared with CTRL. (l, m) No differences were observed between the groups for data from cingulate 1 (l) and cingulate 2 (m) cortex. (n and o) A reduction of GAD-6 immunoreactivity was observed in PPS rats at the level of the medial orbitofrontal (n) and ventral orbitofrontal (o) cortices. (p and q) A tendency toward GAD-6 protein reduction was observed in the lateral orbitofrontal (p) and dorsolateral orbitofrontal (q) cortex of PPS animals in comparison to CTRL rats. Scale bar, 100 μm. *N*: CTRL=8–9, PPS=5–6. *p<0.05, **p<0.01, main effect of stress, results are expressed as the mean±SEM. CTRL, control; PFC, prefrontal cortex; PPS, peripubertally stressed.

GAD-6 immunoreactivity was assessed in several PFC regions (Figure 2). PPS animals exhibited reduced GAD-6 expression across layers throughout the PL (Figure 2j) (*F*_(1,12)_=6.617, *p*=0.024) and IL (Figure 2k) (*F*_(1,11)_=5.512, *p*=0.039) cortices. No differences were observed between the groups throughout the Cg1 (Figure 2l) (*F*_(1,13)_=0.061, *p*=0.809) or Cg2 (Figure 2m) (*F*_(1,13)_=0.552, *p*=0.471). PPS animals had decreased levels of GAD-6 in the MO (Figure 2n) (*F*_(1,13)_=7.131, *p*=0.019) and in VO (Figure 2o) (*F*_(1,13)_=18.509, *p*=0.001). Moreover, PPS animals exhibited a trend toward a reduction in GAD-6 in the LO (Figure 2p) (*F*_(1,13)_=3.746, *p*=0.075) and in the DLO (Figure 2q) (*F*_(1,13)_=3.906, *p*=0.070). Collectively, these results indicate that PPS induces a long-term reduction in GAD in specific PFC subregions.

### PPS Leads to Reduced NLGN-2 mRNA Expression in the PL Cortex

**Figure 3:**
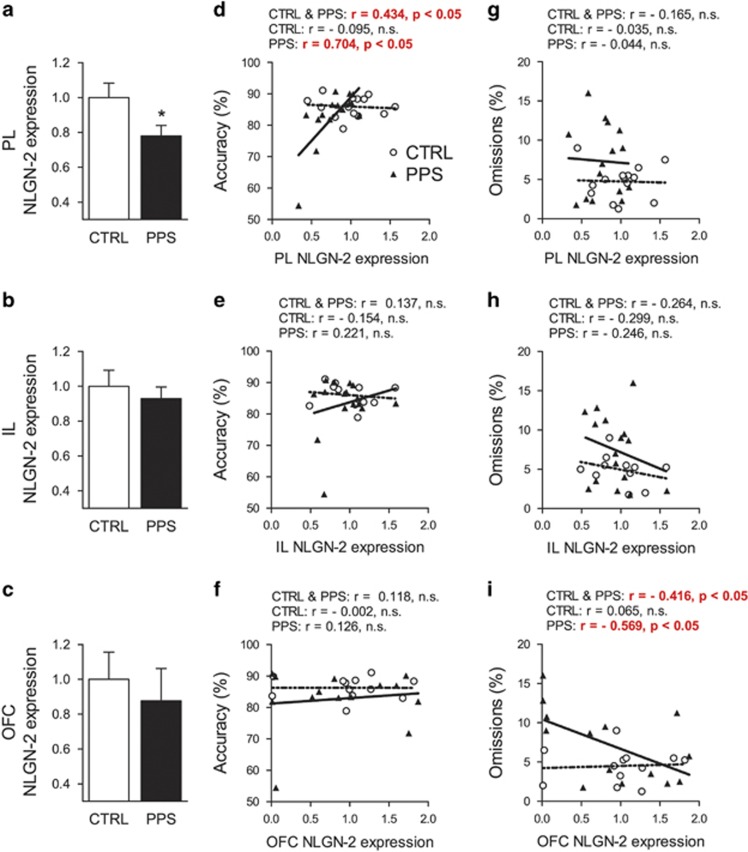
Effects of exposure to peripubertal stress on NLGN-2 gene expression (mRNA) in the prefrontal cortex of adult rats and correlations of gene expression with 5-CSRTT parameters. PPS rats displayed reduced NLGN-2 mRNA expression in the prelimbic cortex (a), while no changes in the infralimbic cortex (b) or the orbitofrontal cortex (c). Significant positive correlations were observed between 5-CSRTT accuracy and NLGN-2 expression in the prelimbic cortex for all animals and for the PPS group (d). No correlations were found between NLGN-2 expression and accuracy in the infralimbic cortex (e) or the orbitofrontal cortex (f). Regarding omissions, while no significant correlations were found with NLGN-2 expression in the prelimbic cortex (g) and infralimbic cortex (h), a significant negative correlation was observed in the orbitofrontal cortex, for data from all animals and for data from only PPS rats (i). *p<0.05, results are expressed as the mean±SEM. 5-CSRTT, five-choice serial reaction time task; CSRTT, choice serial reaction time task; CTRL, control; NLGN-2, neuroligin-2; PPS, peripubertally stressed.

Compared with CTRL rats, PPS animals exhibited reduced NLGN-2 expression in the PL cortex (Figure 3a) (*t*_27_=2.200, *p*=0.037) without significant differences in the IL cortex (Figure 3b) (*t*_25_=0.630, *p*=0.534) or in the OFC (Figure 3c) (*t*_24_=0.499, *p*=0.623). Interestingly, we found a significant positive correlation between PL NLGN-2 expression and accuracy in the 5-CSRTT (Figure 3d) for analyses that included data from all animals (*r*_27_=0.434, *p*=0.019) or only PPS rats (*r*_13_=0.704, *p*=0.003) but not for analyses that only included data for CTRL rats. No correlations between accuracy and NLGN2 in either the IL (Figure 3e) or OFC (Figure 3f) were found. No correlations between omissions and PL NLGN-2 (Figure 3g) or IL NLGN-2 (Figure 3h) were found. Interestingly, omissions in the 5-CSRTT correlated negatively with NLGN-2 expression in the OFC (Figure 3i), which was apparent when considering data from all animals (*r*_24_=−0.416, *p*=0.034) or from the PPS group (*r*_12_=−0.569, *p*=0.034) but not from CTRL rats.

### NLGN-2 OE in the PFC Alleviates Attention Deficits Induced by PPS

**Figure 4:**
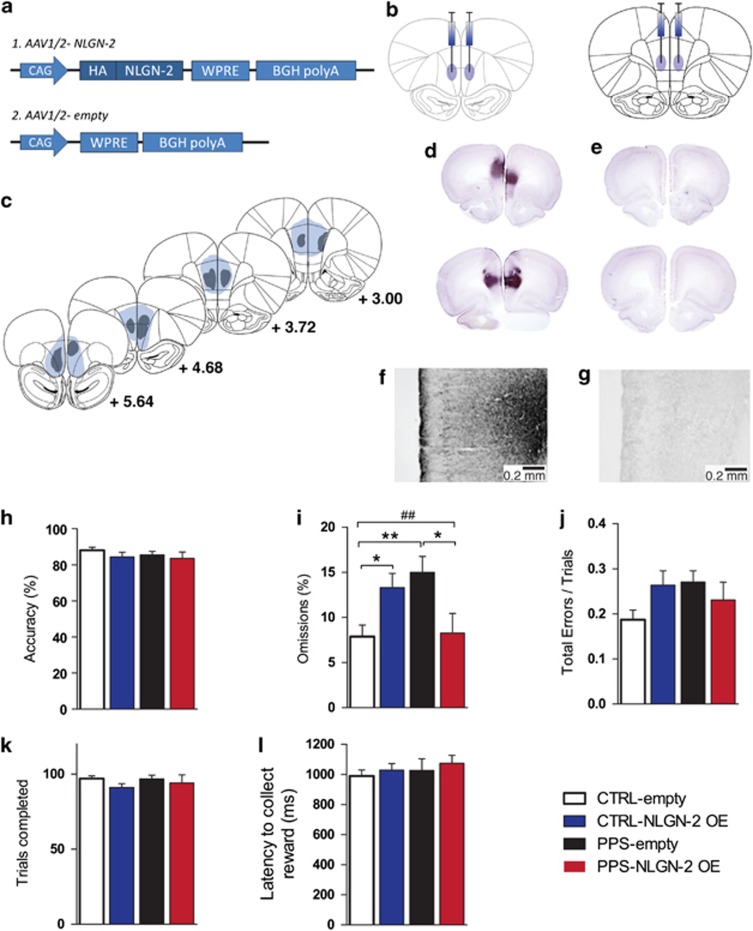
Behavioral effects of NLGN-2 overexpression (OE) in the prefrontal cortex of PPS and CTRL rats under well-trained conditions (baseline) in the 5-CSRTT. (a) Schematic representation of the adeno-associated NLGN-2 expressing virus (1) and the empty viral construct (2). (b) Viral infusion sites in the prefrontal cortex (left: orbitofrontal, right: medial prefrontal cortex). (c) Diagrams of NLGN-2 virus spread derived from the immunohistochemical staining and indicating the maximal (in blue) and minimal (in grey) spread; numbers in illustrations represent the distance from bregma. (d) Representative photomicrographs of the two injection sites (up: orbitofrontal, down: medial prefrontal cortex) where NLGN-2 OE can be observed (immunohistochemical staining). (e) Representative photomicrographs of the two injection sites with the empty viral construct. (f) Higher magnification image of NLGN-2-OE infusion in the prelimbic cortex, and (g) the corresponding higher magnification image of the empty construct infusion. Scale bar 0.2 mm. (h) No difference between the groups was observed in their accuracy in the 5-CSRTT. (i) There was a significant stress × virus interaction for 5-CSRTT omissions. PPS-empty animals performed more omissions than CTRL-empty animals. However, PPS-NLGN-2 OE rats performed fewer omissions than PPS-empty animals. In addition, CTRL-NLGN-2 OE animals performed more omissions than CTRL-empty rats. No difference between the groups was observed for total errors (j), number of trials completed (k), or latency to collect the reward (l), indicating no lack of motivation. *N*: CTRL-empty=9, CTRL-NLGN-2 OE=12, PPS-empty=9, PPS-NLGN-2 OE=9. **p<0.01, *p<0.05 main effect of stress, ^##^*p*<0.01, stress × virus interaction, results are expressed as the mean±SEM. 5-CSRTT, five-choice serial reaction time task; CSRTT, choice serial reaction time task; CTRL, control; NLGN-2, neuroligin-2; PPS, peripubertally stressed.

To directly study the contribution of PFC NLGN-2 expression to the attentional deficits induced by early life stress, we overexpressed NLGN-2 in the PFC at adulthood. AAV-induced NLGN-2 expression colocalizes with gephyrin, which is a post-synaptic molecule expressed at GABAergic neurons (van der Kooij *et al*, 2014). AAV-induced NLGN-2 OE was targeted to the mPFC and OFC subregions (Figure 4a–d). The localization and spread of the virus were confirmed using DAB immunostaining in PFC coronal sections following behavioral testing (Figure 4c–g). As expected, NLGN-2 OE was not observed in animals infused with the empty viral construct (Figure 4e and g). Expression levels measured with quantitative RT–PCR indicated a twofold expression of NLGN-2 in the targeted brain regions (Supplementary Figure S5).

Following a recovery period to allow for the spread of AAV-induced NLGN-2 OE within the targeted PFC areas, adult CTRL and PPS rats infused with either empty or NLGN-2 OE virus were subjected to the 5-CSRTT. As observed in the previous experiment, no differences in the accuracy were observed between the groups (Figure 4h). Importantly, a significant stress × virus interaction was observed for omissions (Figure 4i) (stress × virus interaction: *F*_(1, 35)_=12.333, *p*=0.001), with *post hoc* analyses revealing that PPS-empty animals perform significantly more omissions than CTRL-empty rats (*p*=0.008), as expected from the previous experiment, whereas PPS-NLGN-2 OE animals exhibit a reduction in omissions compared with PPS-empty animals (*p=*0.012). Strikingly, CTRL-NLGN-2 OE animals also performed more omissions than CTRL-empty rats (*p*=0.028). No significant differences were observed between the groups for total errors (Figure 4j), the number of trials completed (Figure 4k), or the latency to collect the reward (Figure 4l), indicating motivation to perform the task. These results support the view that NLGN-2 expression in the PFC plays a causal role in the attentional deficits observed, namely increased omissions, following PPS.

## Discussion

In the present study, we identify the developmental period of peripuberty, comprising childhood and puberty, as a time window of vulnerability to stress, leading to the development of attentional deficits, and reveal novel mechanisms in the PFC in translating these stress effects. Specifically, we highlight NLGN-2 as a critical mediator of PPS detrimental effects on attention, as manifested in increased omissions.

We confirm a link between early life stress and persistent attentional deficits (Carlyle *et al*, 2012; Fuentes *et al*, 2014) and highlight the peripubertal period as a particularly vulnerable phase (Comeau *et al*, 2014). Importantly, deficits in PPS animals were observed both during task acquisition and following substantial training. Together with a delay in acquisition, in the early phases of training, PPS animals exhibited decreased accuracy, an increased number of omissions and a higher number of total errors, which are all indicative of attention deficits. Moreover, well-trained PPS animals continued to exhibit attention deficits as indicated by their higher number of omissions despite lack of changes in accuracy or motivation to perform the task (Amitai and Markou, 2010; Robbins, 2002). Furthermore, PPS animals exhibited an additional striking feature of cognitive dysfunction, ie, high intra-individual variability, which is frequently observed in patients of a wide variety of neuropsychiatric disorders (eg, schizophrenia, bipolar disorder, depression, attention-deficit hyperactivity disorder, and autism) (Di Martino *et al*, 2008; Geurts *et al*, 2008; Kaiser *et al*, 2008; Klein *et al*, 2006). In addition, although indices of impulsivity were not affected in PPS animals, these animals exhibited certain signs of compulsivity. Collectively, the increased omissions, higher variability in the reaction times, increased aspects of compulsive responses, and difficulties in acquisition exhibited by PPS animals indicate a general cognitive deficit, a notion that was also supported by PCA analysis including all relevant 5-CSRTT parameters.

Consistent with the proposed role of inhibitory transmission in the PFC in attentional processes, we found that attentional deficits following PPS are accompanied by a reduction in GAD expression across different layers in the mPFC and in the medial and ventral OFC. These results are consistent with the notion that although contrasting effects are observed in the mPFC and OFC when stress is experienced at adulthood, alterations in these regions generally proceed in the same direction when stress occurs during early development (Liston *et al*, 2006). The functional relevance of these findings, in light of the attention deficits exhibited by PPS rats, is supported by pharmacological studies indicating that the inhibition of GABA synthesis (Asinof and Paine, 2013) or antagonism of GABA_A_ receptors (Paine *et al*, 2011) in the mPFC results in increased omissions in the 5-CSRTT, whereas the inhibition of GAD systemically or within the anterior Cg (which did not exhibit alterations in GAD expression in PPS rats) does not affect the number of omissions (Pehrson *et al*, 2013).

Importantly, we also report that in PPS animals, NLGN-2 expression was downregulated in the PL cortex and positively correlated with accuracy, whereas its expression in the OFC was negatively correlated with omissions. Note that these correlations were found during baseline performance under well-trained conditions, when PPS animals show higher omissions while their accuracy does not differ from controls. Rescuing NLGN-2 expression through AAV genetic targeting in these regions reversed the increased omissions in well-trained PPS animals. Although the lack of effect of NLGN-2 OE on accuracy might seem counterintuitive given the correlation reported for PPS animals between this parameter and NLGN-2 expression in the PL cortex, it is important to note that this correlation was absent in controls in which values of this adhesion molecule were higher. This suggests that a correlational link between NLGN-2 expression in the PL cortex and accuracy might only be revealed under a deficient expression, not OE, of this adhesion molecule. Furthermore, the fact that whereas NLGN-2 expression in the OFC negatively correlated with omissions despite not being significantly different from controls points out at a combined effect of the NLGN-2 OE treatment throughout the targeted brain regions. However, the distributed nature of the NLGN-2 OE in this study precludes from exploring its potential effects when specifically targeting each of the PFC regions separately. It should be noted on the other hand, that NLGN-2 OE in controls led to increased omissions. This observation could suggest that optimal expression values of this adhesion molecule in the PFC can be important for accurate performance. This is in line with the view that altered balance in the expression of molecules necessary for synapse specification, such as NLGNs, can lead to deficits in circuit function and behavioral control (Dahlhaus *et al*, 2010). However, a note of caution should be drawn before future studies confirm this conclusion, as the quantitative validation of NLGN-2 expression in our study was performed in controls, not in peripubertally stressed animals.

Overall, these results are consistent with recent evidence suggesting that NLGN-2 may represent a relevant target of stress. Specifically, a reduction in NLGN-2 expression, concurrent with alterations in social behaviors, was reported in the hippocampus following exposure to chronic restraint stress at adulthood (van der Kooij *et al*, 2014). In contrast, genetic OE of NLGN-2 was shown to increase GAD levels (Kohl *et al*, 2013) and to reduce the excitation and inhibition ratio in the frontal cortex (Hines *et al*, 2008). Conversely, NLGN-2 genetic deletion reduced inhibitory synaptic function (Blundell *et al*, 2009).

To date, the majority of studies revealing a role for NLGNs in brain function and cognition have been of genetic origin. On the one hand, mutations in NLGN-encoding genes have been associated with cognitive diseases such as autism and schizophrenia. On the other hand, mice genetically modified for NLGN genes have confirmed the impact of these molecules in specific aspects of synaptic function and social behaviors (Hines *et al*, 2008; Kohl *et al*, 2013; Wohr *et al*, 2013; Kohl *et al*, 2015). Our findings strongly support a role for NLGN in cognition. Moreover, the apparently paradoxical observation of impaired attention in control animals overexpressing NLGN-2 suggests that the disruption of optimal NLGN-2 levels may result in negative consequences for cognitive performance, which is consistent with pharmacological studies demonstrating attention deficits following either activation or inhibition of PFC GABAergic function from normative levels (Pezze *et al*, 2014).

Given the link between NLGN gene mutations and certain developmental disorders (Sudhof, 2008), our finding that early stress can affect neurodevelopmental trajectories involving lasting alterations in NLGN-2 expression suggests that stress may affect specific cognitive functions by acting on mechanisms that are similar to those targeted by genetic disorders. Moreover, as patients with neurodevelopmental disorders (eg, autism, schizophrenia) generally exhibit enhanced emotional vulnerability (Mazefsky *et al*, 2013), stress may act as an active contributing factor to the development of the disorder. Accordingly, our results highlight NLGN-2 as a relevant target for future preclinical studies that combine animal models of neurodevelopmental disorders (eg, autism, schizophrenia) with early life stress. Our results also support the interest in developing research programs to specifically examine cognitive outcomes in NLGN-2 mutant mice subjected to early life stress.

An additional interesting implication derived from the observation that OE of NLGN-2 at adulthood alleviated the primary cognitive deficit elicited by PPS suggests that certain deficits induced by early stress are reversible even after the critical developmental window. Although the current success of therapies involving gene delivery into the brain have encountered technical and safety challenges, considerable ongoing progress in the field offers optimistic prospects for the use of CNS gene therapy in the near future (Aubourg, 2013). Alternatively, NLGN-2 function may be more easily targeted using pharmacological approaches based on recently developed peptide strategies (Berezin and Bock, 2010). Recently, we demonstrated that treatment with a synthetic peptide that comprises NLGN-2 sequences that interact with its presynaptic partner neurexin-1 efficiently and specifically modulated social behaviors (van der Kooij *et al*, 2014).

Therefore, the present study delineates GABAergic dysfunction in the PFC as a long-term consequence of early life stress and critically identifies NLGN-2 as a novel promising target to reverse stress-induced attention deficits, particularly omissions.

## Funding and Disclosure

This work was supported by grants from the Swiss National Science Foundation (31003A-152614 and the NCCR Synapsy), the EU FP7 project MATRICS (no 603016), Oak Foundation, and intramural funding from the EPFL to CS and Spanish Ministry of Economy and Competitiveness BFU2012-32512, Generalitat Valenciana ACOMP/2012/229, and Prometeo Excellence Program PROMETEO2013/069 and the Fundación Alicia Koplowitz to JN.
