# Supplementary material for: Neuroligin-2 Expression in the Prefrontal Cortex is Involved in Attention Deficits Induced by Peripubertal Stress
Source: Neuropsychopharmacology. 2015 Aug 5;41(3):751–61. doi: 10.1038/npp.2015.200 (PMC4707821; doi:10.1038/npp.2015.200)
Supplement: Supplementary Information [file npp2015200x1.pdf]

Supplementary information to:

Neuroligin-2 expression in the prefrontal cortex is involved in attention deficits induced by peripubertal stress

Stamatina Tzanoulinou, Clara García-Mompó, Orbicia Riccio, Jocelyn Grosse, Olivia Zanoletti, Panagiotis Dedousis, Juan Nacher and Carmen Sandi

Supplementary Materials and Methods

Peripubertal Stress Protocol

The stress protocol consisted of presenting two different fear-inducing stressors (each one lasting 25 min): (1) exposure to the synthetic fox odor trimethylthiazoline (9 μl) (Phero Tech Inc., Delta, BC, Canada) released through a small cloth, in a plastic box (38 cm length, 27.5 cm width and 31 cm height) placed under a bright light (210–250 lx); and (2) exposure to an elevated platform (12 × 12 cm, elevated 95 cm from the ground) under direct bright light (470–500 lx). Following each stress session, the animals were returned to their home cages where, for 15 min, a transparent Plexiglas wall with holes separated each animal. The stressors were applied during the peripubertal period (a total of 7 days across postnatal day P28 to P42, i.e., on P28–P30, P34, P36, P40 and P42); during the light phase; and according to a variable schedule. The order and timing of the stressors were changed on different days. On some stress days, only one stressor was presented, while on other days, the two stressors were given consecutively. The control animals were handled on the days that their experimental counterparts were exposed to stress. Following each stress session, the animals remained separated for 15 minutes before rejoining their cage mates. Animals in the same cage were always assigned to the same experimental group (either CTRL or PPS).

Five-Choice Serial Reaction Time Task (5-CSRTT)

The protocol flow and task contingencies were controlled using GraphicState 3.03 software and the data analysis was made using a custom-made Visual Basic for Applications code running in Microsoft

Excel sheets. Following the assessment of the rats' baseline performance, they were subjected to the impulsivity screening procedure which involved increasing the inter-trial interval (ITI) from 5 to 7 seconds for 3 weeks. This manipulation was performed only once per week preceded and followed by 2 days of normal ITI duration and has been shown to increase the impulsivity responding of the animals (McNamara *et al*, 2010).

In the NLGN-2 OE experiment, two animals (1 PPS-empty and 1 PPS-NLGN-2 OE) were excluded from the analyses as their performance was below criterion (performing fewer than 10 trials per session). In addition, given the very lengthy training involved in the standard 5-CSRTT protocol (note that animals in the first experiment required between 3 and 8 weeks to go through all training stages and proceed to the baseline testing) and the time constraints derived from analyzing animals submitted to AAV-induced brain region-specific gene overexpression, behavior of the animals for the well-trained phase was assessed at an earlier stage than usually (stage 7), still a rather challenging stage. In addition to virus-related timing issues, this time point was selected based on the results obtained during the first experiment.

GAD-6 immunohistochemistry and quantification of neuropil immunoreactivity

For the GAD-6 immunohistochemistry experiment the rats were anesthetized with a lethal dose of pentobarbital (Esconarkon, Streuli Pharma AG, 150 mg/kg body weight, solution provided by the EPFL veterinarian) and transcardially perfused as previously described (Kohl *et al*, 2013).

Subseries of 50 µm thick sections from each group of animals were processed free floating for immunohistochemistry using the avidin-biotin-peroxidase (ABC) method (Hsu *et al*, 1981). Sections were first incubated for 1 minute in an antigen unmasking solution (0.01 M citrate buffer, pH 6) at 100°C. After cooling the sections down to room temperature, they were incubated with 3% H_2O_2 in phosphate buffered saline (PBS) for 10 minutes to block endogenous peroxidase activity. They were then treated for 1 hour with 10% normal donkey serum (NDS) (Jackson ImmunoResearch

Laboratories) in PBS with 0.2% Triton-X100 (Sigma-Aldrich) and incubated for 24 hours at room temperature in the primary antibody anti-GAD-6, generated in mouse, (DSHB, 1:500) with PBS containing 0.2% Triton-X-100 and 5% NDS. On the second day, sections were incubated for 1 hour at 25°C with the biotinilated secondary antibody: donkey anti-mouse IgG (Jackson ImmunoResearch Laboratories, 1:200), followed by an avidin-biotin-peroxidase complex (ABC; Vector Laboratories) for 30 minutes in PBS. Color development was achieved by incubating with 3,3'-diaminobenzidine tetrahydrochloride (DAB; Sigma-Aldrich) and 0.033% H_2O_2 for 4 minutes. Finally, sections were mounted on slides, dried for one day at room temperature, dehydrated with ascending alcohols and rinsed in xylene. Sections were coverslipped using Eukitt mounting medium (PANREAC). All studied sections passed through all procedures simultaneously in order to minimize any difference from the immunohistochemical staining itself. To avoid any bias in the analysis, all slides were coded prior to analysis and remained so until the experiment was completed. A Nissl staining (toluidine blue) in alternate series of sections was used for determining layer and area boundaries within mPFC regions and OFC regions, based on cytoarchitectural differences across these layers and areas (Figure S4). Sections were examined with an Olympus CX41 microscope under bright-field illumination, homogeneously illuminated and digitalized using a CCD camera. Photographs of the different areas were taken at 20× magnification. Grey levels were converted to optical densities (OD) using Image J software (NIH). Means were determined for each experimental group and data were analyzed by repeated measures ANOVA.

Viral Overexpression of NLGN-2

The vector incorporated the following regulatory elements: rAAV2 inverted terminal repeat (ITR) sequences, a scaffold attachment region (SAR) element, the hybrid chicken B-actin/CMV enhancer (CAG) promoter region, a cis-acting woodchuck post-transcriptional regulatory element (WPRE) and a bovine growth hormone polyadenylation signal sequence (bgh-polyA) (Figure 4A). The mouse pCAG-HA-tagged-NLGN-2 plasmid was kindly provided by Prof. Dr. Peter Scheiffele, University of Basel, Switzerland. For the empty vector, the same backbone without the cDNA was used. Initially, animals

were anaesthetized with an intraperitoneal injection of ketamine (75 mg/kg body weight) and xylazine (10 mg/kg body weight) and installed in a stereotactic frame to avoid any head movements during the surgical procedure. A total of 3 µl of either NLGN-2-OE or empty vector (titres: NLGN-2-OE: 1.4 × 10^{12}-diluted 4 times- genomic particles/ml; empty: 1.3 × 10^{12} - diluted 4 times - genomic particles/ml) was bilaterally injected (two injection sites per hemisphere, 1.5 µl each) in the orbital and medial prefrontal cortex (Figure 4B) using automated syringe pumps with a flow rate of 0.2 µl/min. The injectors were left in site for 5 additional minutes after the actual injection. After removing the injectors, animals were injected with an antisedant (0.1 mg/kg of body weight, Antisedan, Pfizer, Switzerland). All rats were treated with paracetamol (500 mg/700 ml H_2O, Dafalgan, Bristol-Myers Squibb, Agen, France) via the drinking water for 7 days after the surgery. The animals were allowed to recover for 4 weeks (allowing also for sufficient NLGN-2 overexpression) from surgery before starting behavioral testing. In order to verify the coordinates and overexpression of the virus, immunohistochemistry for NLGN-2 was performed. The conditions and protocol were performed as previously described (Kohl *et al*, 2013). In brief, after incubation in 0.3% H_2O_2/PBS to block endogenous peroxidases, the floating sections were blocked in 10% donkey serum/PBS-T and incubated overnight with the primary antibody against NLGN-2 (1:6000, Synaptic Systems) at 4^oC. Sections were rinsed in PBS-T and then incubated with the secondary antibody (biotinylated anti-rabbit IgG, 1:2000, Vector Laboratories, USA) for 2 hours. Afterwards sections were treated using an ABC kit (Vectastain ABC Kit, Vector Laboratories, USA), followed by further washes in PBS until color development using 3, 39- diaminobenzidine (DAB substrate kit for peroxidase, Vector laboratories). Images were taken with a bright field microscope of 1.25× objective for whole brain images and of 10× objective for PFC images.

Statistics

Survival analyses were performed according the Kaplan-Meier method and the log rank results are reported. Regarding t-tests, if Levene's test for equality of variances was significant, equal variance

was not assumed and the altered degrees of freedom were rounded to the nearest whole number. Regarding ANOVAs, if Mauchly's test of sphericity was significant, the Greenhouse-Geisser correction is reported along with the corresponding adjusted *F*, *p* and *df* values. In case of significant interactions revealed by the two-way ANOVA, follow-up analyses were performed with the LSD post hoc test. In order to investigate possible relationships between NLGN-2 mRNA expression in the prefrontal cortex and performance in the 5-CSRTT, correlational analyses were performed. Specifically, the NLGN-2 expression in the different PFC regions was correlated with two main 5-CSRTT parameters, accuracy and omissions, considering both groups and then examining each group separately. Pearson's *r*, *df* and the significance are reported regarding the correlations.

Principal Components Analysis

Principal Components Analysis was applied to explore relationships of basic 5-CSRTT parameters and subsequently to compare CTRL and PPS rats in the derived factors. For the factorial analysis, the number of extracted factors was not pre-defined. Rather, PCAs were applied to selected parameters from the aforementioned test. A continuous, interval scale score was calculated for each factor using principal components as the extraction method and varimax rotation with Kaiser normalization rotation (Doremus *et al*, 2006). Then, individual factor scores were calculated for each subject through the relative weight and orientation (eigenvalues) of the parameters for each factor. Scores were generated using a Z distribution, where the value 0 corresponds to the mean, and values are expressed in terms of standard deviations. Subsequently the mean scores of the animals were plotted for CTRL and PPS groups and compared using t-tests.

Supplementary Results

Peripubertal stress delays the acquisition of the 5-CSRTT

During the training phase, it was evident that PPS animals performed more total errors (omissions and incorrect responses) than CTRL rats throughout the acquisition phase (Figure S1A) (stress × time

interaction: $F_{(4, 110)} = 1.048$, $p = 0.385$, main effect of stress: $F_{(1, 28)} = 4.359$, $p = 0.046$), which also holds independently for the first block of 5 sessions (stress × time interaction: $F_{(2, 65)} = 1.137$, $p = 0.333$, main effect of stress: $F_{(1, 28)} = 4.945$, $p = 0.034$). No difference was observed between PPS and CTRL rats in their total trials completed, indicating no apparent differences in their motivation to perform the task (stress × time interaction: $F_{(5, 132)} = 0.760$, $p = 0.573$, main effect of stress: $F_{(1, 28)} = 2.247$, $p = 0.145$, data not shown). Interestingly, a survival analysis revealed that PPS animals required significantly more days than CTRL rats in order to progress throughout the different training stages, which was statistically significant on accumulated data till stage 6 (Figure S1B) ($\chi^2_{(1)} = 4.326$, $p = 0.038$). Survival analysis of all the days needed to reach the most advanced stage (stage 12) did not reveal significant differences between the groups ($\chi^2_{(1)} = 2.290$, $p = 0.130$, data not shown). Overall, these results indicate that peripubertally stressed animals perform suboptimally during the initial training phase in the 5-CSRTT compared to CTRL rats, showing a delayed acquisition pattern that was particularly marked during the first 5 sessions of training.

Peripubertally stressed rats show an overall suboptimal performance in the 5-CSRTT according to principal components analysis-derived factors

In order to investigate possible underlying relations between the various 5-CSRTT parameters, we performed a principal component analysis (PCA) including the most relevant parameters measured under baseline conditions. This analysis produced a solution with 2 factors (Figure S2A). The first factor contained loadings of omission and commission errors (total errors), latency to respond correctly, correct responses, as well as variability of reaction time for correct responses. Thus, the first factor was considered to represent an index of general performance. The exact relationships between these variables, as well as their loading values are listed in Figure S2A. Parameters that concerned premature responses and continuous hits in the magazine were loaded into the second factor that was, therefore, considered to represent an impulsivity/compulsivity index. These two factors could explain 78.5 % of the total variance. The scores for each animal in these factors were

subsequently calculated and compared between the groups. PPS rats scored higher on the general performance index, therefore performing significantly poorer in the 5-CSRTT, given that correct responses have a negative sign in the PCA (Figure S2B) (t_{18} = -2.520, p = 0.022) but no significant differences were observed between the groups for the impulsivity/compulsivity factor (Figure S2C) (t_{28} = -1.031, p= 0.312).

Peripubertally stressed animals do not show increased impulsivity upon prolongation of the inter-trial interval in the 5-CSRTT

In order to explicitly examine whether peripubertal stress had affected impulsivity behavior in the 5-CSRTT, we increased the inter-trial interval from 5 to 7 seconds. No difference was observed between PPS and CTRL rats in any of the sessions across the three weeks when this manipulation was performed (Figure S3) (week 1: t_{28} = -1.001, p = 0.325, week 2: t_{27} = 0.672, p = 0.507, week 3: t_{27} = 1.416, p = 0.168).

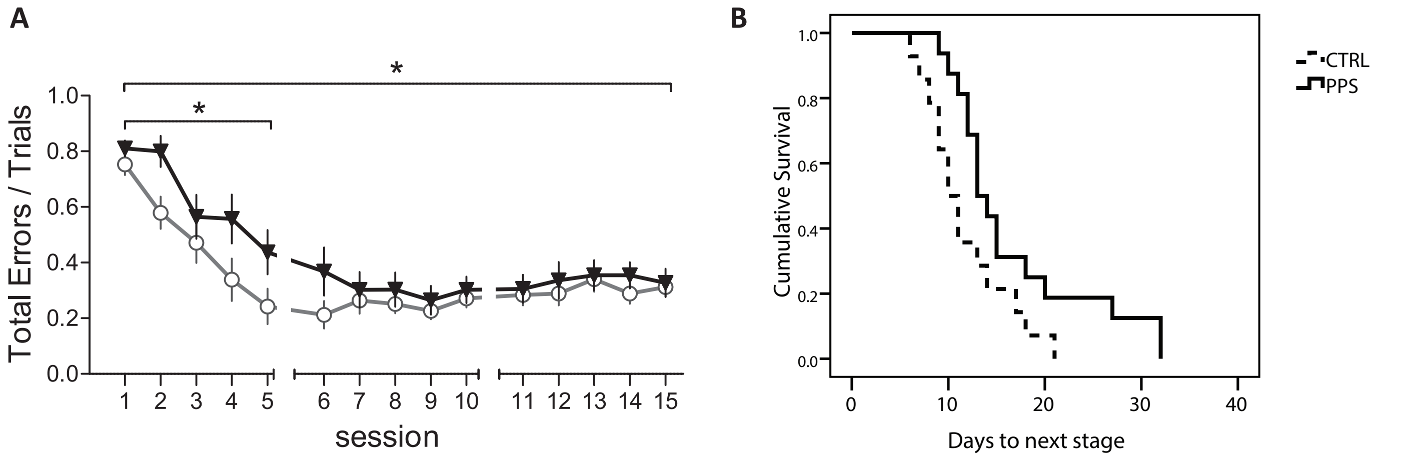

Figure S1. Peripubertal stress effects on the acquisition of the 5-CSRTT at adulthood. **A**, Across the 15 initial training sessions, peripubertally stressed animals performed more total errors, normalized by the number of total trials, compared to CTRL rats. Block-wise, this effect was significant for the first 5-session block. **B**, Survival analysis revealed that PPS animals needed significantly more days to reach the criteria for moving to the next stage until stage 6. (N: CTRL = 14, PPS = 16), * $p < 0.05$ main effect of stress, results are expressed as the mean ± S.E.M.

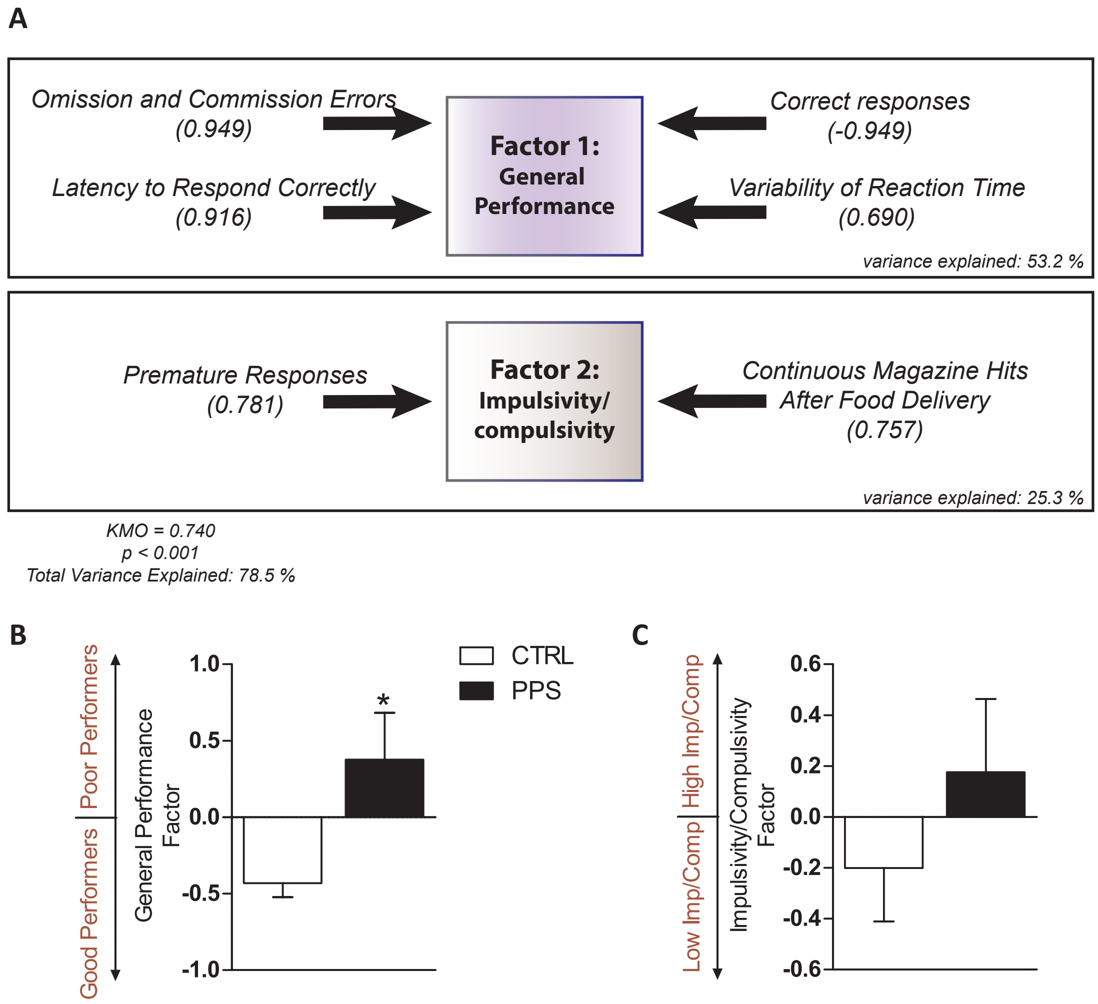

Figure S2. Principal component analysis (PCA) of the 5-CSRTT variables measured at baseline conditions. **A**, Two factors were derived for both groups of rats. Factor 1 consisted of loadings of total errors, correct responses, latency to respond correctly and the intra-individual variability of reaction time, thus reflecting general performance. Factor 2 consisted of premature responses and repetitive responding in the food magazine, thus representing impulsivity/compulsivity aspects. Factor 1 explained 53.2% of the variance, whereas Factor 2 explained 25.3%. **B**, Comparison of Factor 1 scores between the groups revealed that peripubertally stressed animals scored higher in this factor compared to CTRL rats, thus indicating poor performance. **C**, No difference was observed between PPS and CTRL rats regarding Factor 2 scores.* $p < 0.05$ vs. CTRL, results are expressed as the mean ± S.E.M.

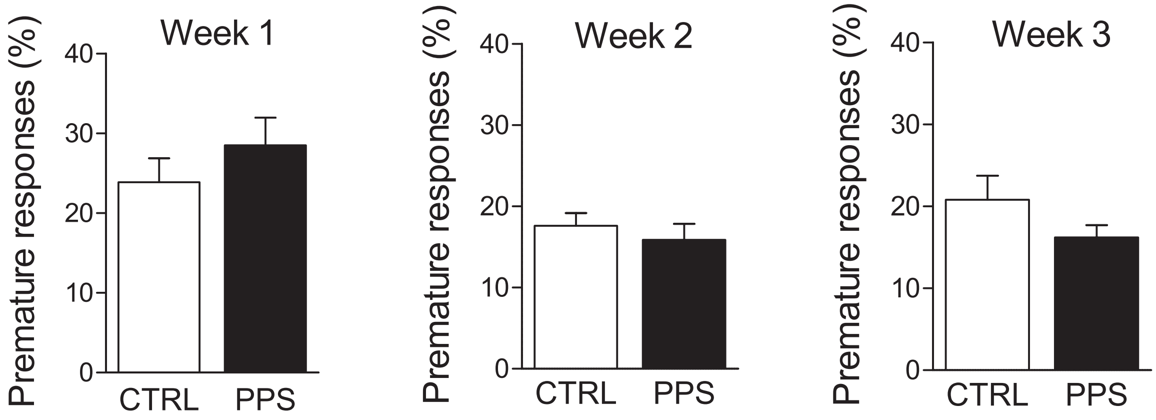

Figure S3. No differences were observed between PPS and CTRL animals when the inter-trial interval was increased from 5 to 7 seconds at any of the three screening weeks. (N: CTRL = 14, PPS = 16), results are expressed as the mean ± S.E.M.

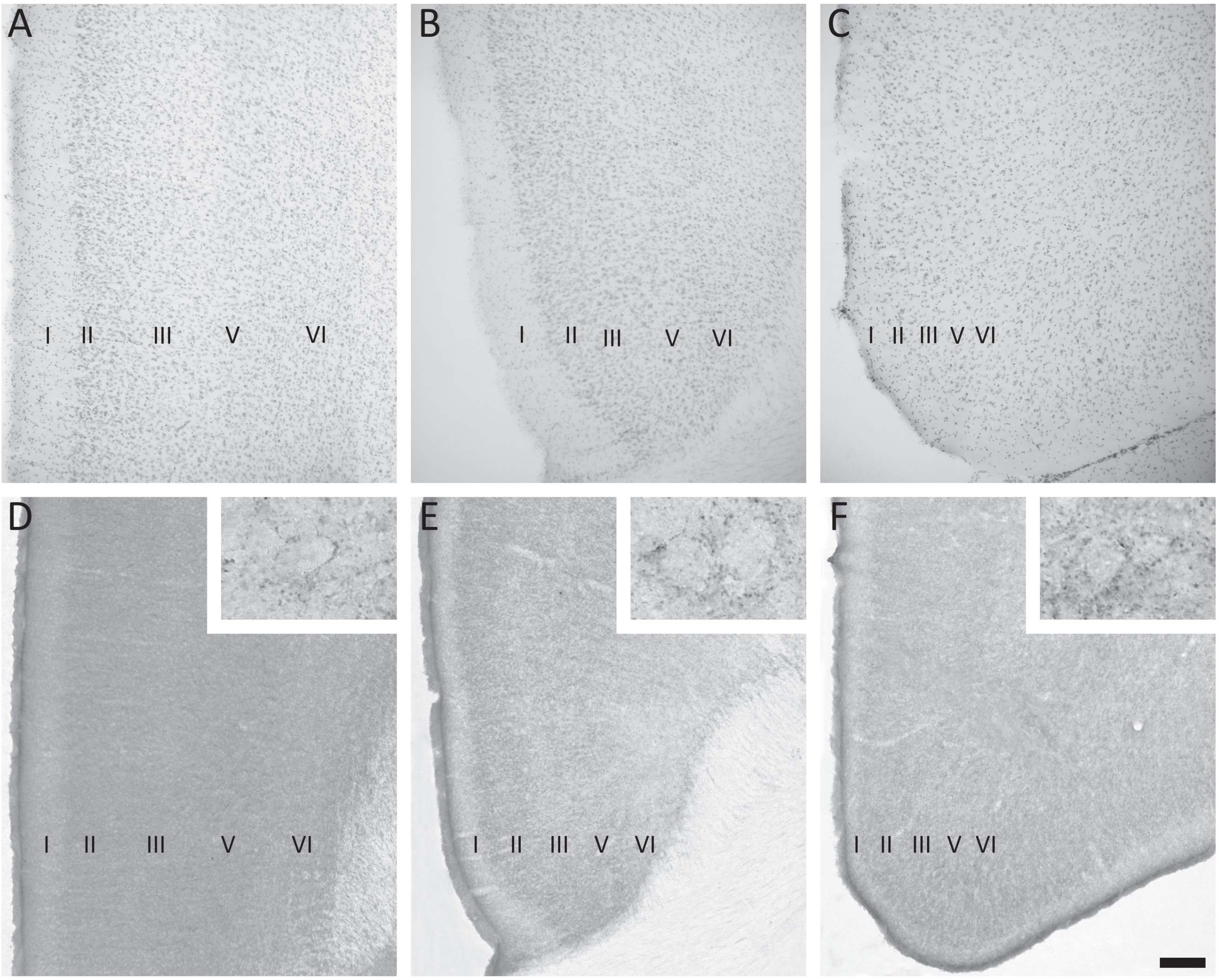

Figure S4. Panoramic view of the rat infralimbic (IL) and prelimbic cortex (PL) (**A**), cingulate cortex, area1 (Cg1), cingulate cortex, area 2 (Cg2) (**B**) and orbitofrontal cortex (OFC) (**C**) stained with toluidine blue. Distribution of GAD-6 inmunoreactivity in the neuropil of the IL and PL (**D**), Cg1, Cg2 (**E**) and OFC cortex (**F**). Detailed view of GAD 6 immunoreactivity in layer III of the IL and PL (**inset in D**), Cg (**inset in E**) and OFC (**inset in F**). Scale bar: 120 µm (A-F) and 20 µm (inset in D, E and F).

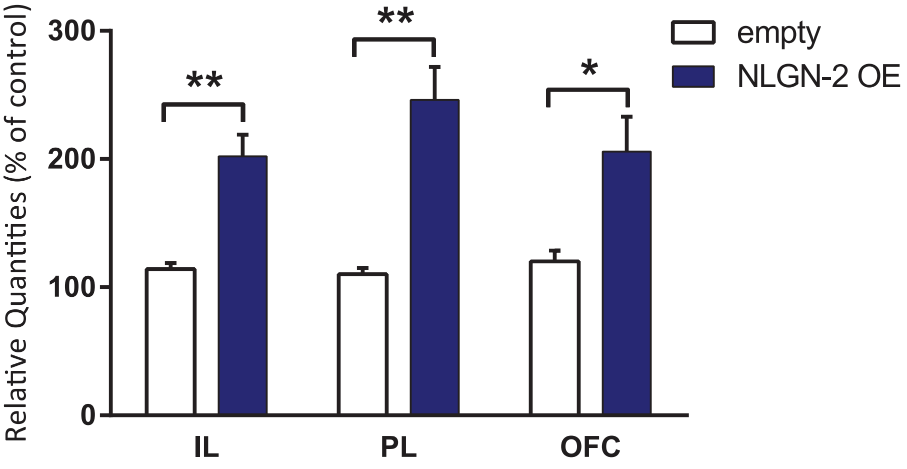

Figure S5. NLGN-2 mRNA is increased in the PFC regions infused with the NLGN-2 overexpressing viral construct. In order to validate the expression levels of NLGN-2 in a quantitative manner we performed quantitative RT-PCR on tissue punches of the regions where the empty or the NLGN-2 OE construct were infused (i.e., infralimbic cortex, prelimbic cortex and orbito-frontal cortex). Indeed, NLGN-2 mRNA was increased in the targeted regions (IL: $t_9 = 4.533$, $p = 0.001$, PL: $t_9 = 4.746$, $p = 0.001$, OFC: $t_9 = 2.751$, $p = 0.022$). (N: empty = 5, NLGN-2 OE = 6), ** $p < 0.01$, * $p < 0.05$, results are expressed as the mean ± S.E.M.

Supplementary References

Doremus TL, Varlinskaya EI, Spear LP (2006). Factor analysis of elevated plus-maze behavior in adolescent and adult rats. Pharmacol Biochem Behav **83**(4): 570-577.

Hsu SM, Raine L, Fanger H (1981). Use of avidin-biotin-peroxidase complex (ABC) in immunoperoxidase techniques: a comparison between ABC and unlabeled antibody (PAP) procedures. *J Histochem Cytochem* **29**(4): 577-580.

Kohl C, Riccio O, Grosse J, Zanoletti O, Fournier C, Schmidt MV*, et al* (2013). Hippocampal neuroligin-2 overexpression leads to reduced aggression and inhibited novelty reactivity in rats. *PloS One* **8**(2): e56871.

McNamara R, Dalley JW, Robbins TW, Everitt BJ, Belin D (2010). Trait-like impulsivity does not predict escalation of heroin self-administration in the rat. *Psychopharmacology* 212(4): 453-464.
